# Welcoming new Editors

**DOI:** 10.1242/dmm.050368

**Published:** 2023-07-18

**Authors:** Kirsty M. Hooper

**Affiliations:** The Company of Biologists, Bidder Building, Station Road, Histon, Cambridge CB24 9LF, UK

**Figure:**
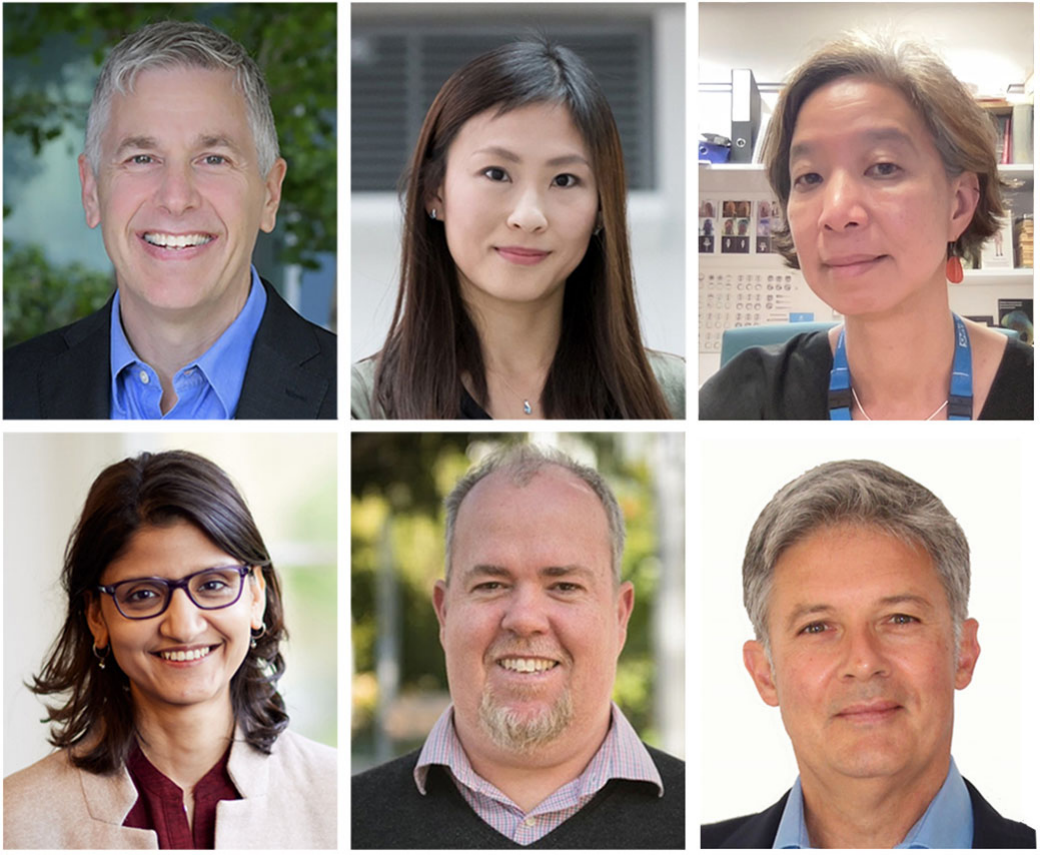
James Amatruda, Vivian Li and Karen Liu (top row, from left to right); Sumana Sanyal, Ian Smyth and Eckhard Wolf (bottom row, from left to right).

Disease Models & Mechanisms (DMM) publishes cutting-edge disease-focused research that aims to bridge the gap between fundamental biology and clinical practice. To help us achieve this, a key aim of our Editor-in-Chief, Liz Patton, is to expand our Editor team and thus broaden our core expertise (Patton, 2021). By building a multidisciplinary team, we have strengthened our roots in a wide range of research fields that impact human disease.

Over the past 2 years, we have welcomed James Amatruda (Children's Hospital Los Angeles, USA), Vivian Li (Francis Crick Institute, UK), Karen Liu (King's College London, UK), Sumana Sanyal (University of Oxford, UK), Ian Smyth (Monash University, Australia) and Eckhard Wolf [Ludwig Maximilian University of Munich (LMU Munich), Germany], all of whom contribute a wealth of knowledge and experience. As active researchers and experts in their respective fields, all our Editors look forward to handling your next submission.

## James Amatruda

James Amatruda is a clinical scientist who specialises in childhood solid tumours, such as sarcomas, germ cell tumours and Wilms tumour. Jim received his MD and PhD degrees from Washington University, prior to a residency at Brigham and Women's Hospital and a Medical Oncology Fellowship at the Dana-Farber Cancer Institute. He established his independent laboratory in 2005 in the Departments of Pediatrics and Molecular Biology at UT Southwestern Medical Center. In 2019, he moved to Children's Hospital Los Angeles, where he is now Head of Basic and Translational Research in the Cancer and Blood Disease Institute, and Professor of Pediatrics and Medicine at the University of Southern California Keck School of Medicine. His research harnesses the strength of zebrafish modelling to translate pathomechanistic findings into diagnostic, prognostic and therapeutic impact in the clinic. He brings a strong clinical focus to the team, and his perspective and experience have been integral to DMM's aim to better connect patient needs with basic and preclinical research.

## Vivian Li

Vivian Li investigates how stem cells maintain healthy organs, with a particular focus on the bowel, associated cancers and Wnt signalling. Vivian studied molecular biotechnology at the Chinese University of Hong Kong, then pursued a PhD in pathology at the University of Hong Kong. She became proficient in organoid research while doing a Croucher Foundation Fellowship in Hans Clevers' laboratory at the Hubrecht Institute in the Netherlands. She then established her laboratory at the Medical Research Council National Institute for Medical Research in London in 2013, and, since 2015, has been group leader of the Stem Cell and Cancer Biology Laboratory at the Francis Crick Institute in London. She recently became Assistant Research Director at the Crick. Her innovative use of human stem cells and organoids will keep DMM closely connected to advances in the *in vitro* modelling of disease.

## Karen Liu

Karen Liu is a developmental biologist with a focus on neural crest development and associated disorders, such as craniofacial anomalies, neuroblastoma and ciliopathies. Karen received an undergraduate degree from Columbia College and did her PhD studies with Richard Harland at the University of California, Berkeley. She then trained with Mike Longaker and Jerry Crabtree at Stanford University, before, in 2007, launching her independent laboratory at King's College London, where she is currently Professor of Genetics and Development in the Centre for Craniofacial and Regenerative Biology. Her work with human stem cells, *Xenopus* and mice builds an in-depth view of the developmental mechanisms that can go awry in congenital disorders. Her knowledge and expertise have been instrumental in launching our 2024 Special Issue entitled ‘Translating Multiscale Research in Rare Disease’.

## Sumana Sanyal

Sumana Sanyal has made key discoveries interrogating the mechanisms of viral pathogenesis and host evasion, with her primary research focus being Zika and dengue viruses, although her laboratory has more recently investigated severe acute respiratory syndrome coronavirus 2 (SARS-CoV-2) and influenza viruses. Sumana received her PhD from Cornell University as part of the Cornell–Rockefeller–Sloan Kettering Tri-Institutional Program, and did her postdoctoral training with Hidde Ploegh at the Whitehead Institute for Biomedical Research at Massachusetts Institute of Technology. She then held a Croucher-Foundation-sponsored Assistant Professor position at the School of Public Health and School of Biomedical Sciences at the Faculty of Medicine, University of Hong Kong. She is currently Associate Professor at the Dunn School of Pathology, University of Oxford and a Wellcome Trust Investigator. Sumana's valuable insight into the field of virology helps DMM support this vital and prominent area of research.

## Ian Smyth

Ian Smyth studies the mechanisms that govern kidney and skin development, primarily in mice, and has advanced our understanding of the genomic and environmental factors driving kidney disorders. Ian's doctoral studies were at the University of Queensland, prior to postdoctoral training as a Wellcome Trust Traveling Research Fellow at the MRC Human Genetics Unit in Edinburgh, then at Baylor College of Medicine in Houston and, finally, as a London Research Institute Fellow at Cancer Research UK, London. In 2006, he started his own laboratory in Melbourne. He is currently the Associate Dean for Research and Research Infrastructure in the Faculty of Medicine, Nursing and Health Sciences at Monash University, and the Head of the Kidney Development and Disease Group at the Monash Biomedicine Discovery Institute. Ian's extensive experience enriches our coverage of kidney disorders, but also helps us support functional genomic disease research in general.

## Eckhard Wolf

Eckhard Wolf is a veterinary scientist and an expert in large-animal modelling of diseases, such as diabetes mellitus, rare monogenic diseases, and kidney and heart disease. Eckhard studied veterinary medicine and received his PhD from LMU Munich, Germany. He continued his postdoctoral training at LMU Munich, before becoming a group leader at the University of Veterinary Sciences, Vienna, Austria. He has since returned to LMU Munich, acting as Head of the Institute for Molecular Animal Breeding and Biotechnology, then Director of the Laboratory for Functional Genome Analysis, and, since 2014, he has been the Director of the Centre for Innovative Medical Models. His pioneering work, particularly with pig models of disease, has helped lead to recent success in heart xenotransplantation, which was highlighted in our recent Special Issue, ‘Moving Heart Failure to Heart Success’ (Wolf et al., 2023). As large animals are essential to recapitulate certain areas of disease biology more faithfully and to test therapies prior to clinical trials, Eckhard's extensive knowledge of translational large-animal models is invaluable to DMM.
